# Trauma and the default mode network: review and exploratory study

**DOI:** 10.3389/fnbeh.2024.1499408

**Published:** 2024-12-23

**Authors:** Aldrich Chan, Philip Harvey, Rene Hernandez-Cardenache, Noam Alperin, Sang Lee, Christopher Hunt, Nick Petersen, Georg Northoff, Nadine Robertson, Jason Ouyang, Ryan Karasik, Kate Williams

**Affiliations:** ^1^Center for Neuropsychology and Consciousness, Miami, FL, United States; ^2^Leonard Miller School of Medicine, University of Miami, Miami, FL, United States; ^3^Graduate School of Education and Psychology, Pepperdine University, Los Angeles, CA, United States; ^4^Center of Excellence in Stress and Mental Health (CESAMH), University of California, San Diego, La Jolla, CA, United States; ^5^Department of Sociology, University of Miami, Miami, FL, United States; ^6^Department of Psychiatry, University of Ottawa, Ottawa, ON, Canada; ^7^Department of Psychiatry and Behavioral Neurobiology, University of Alabama, Birmingham, AL, United States

**Keywords:** trauma-exposed, non-trauma-exposed, default mode network, resilience, trauma, review, PTSD, neuropsychology

## Abstract

While PTSD continues to be researched in great depth, less attention has been given to the continuum of traumatic responses that resides outside this diagnosis. This investigation begins with a literature review examining the spectrum of responses through the lens of the default mode network (DMN). To build upon this literature, a systematic exploratory study was incorporated, examining DMN-related neuropsychological functioning of 27 participants (16 trauma-exposed, and 11 non-trauma-exposed), with a subset (15 participants) completing neuroimaging. This study revealed that in comparison to the control group, the trauma-exposed group had reductions in their capacity for self-referential processing, social cognition, autobiographical recall, prospection, and increased mind-wandering. While correlations were encountered between cognitive findings and brain volume, comparative volumetric findings between trauma-exposed and non-t rauma exposed were insignificant. This suggests that the conservation of DMN structural integrity may play a role in resilience, supporting the existing theory that reduced hippocampal volume may be a pre-existing vulnerability to PTSD rather than a consequence and that reductions in DMN related cognition are functionally mediated.

## Introduction

Greater than 70% of the global population is estimated to experience a traumatic event in their lives (Benjet et al., 2016), yet not all of those exposed to a traumatic event will develop PTSD. There resides a spectrum, with some individuals continuing through life with sub-clinical symptoms in the absence of any formal diagnosis, and others maintaining a level of healthy functioning (Bolsinger et al., 2018). These cases where *covert* traumatic symptoms reside remain understudied, and importantly, the potential protective attributes they possessed may be overlooked. An important question that follows is how do these trauma-exposed (TE) individuals differ when compared to groups of individuals who have not experienced a traumatic event and those with PTSD?

**Figure 1:**
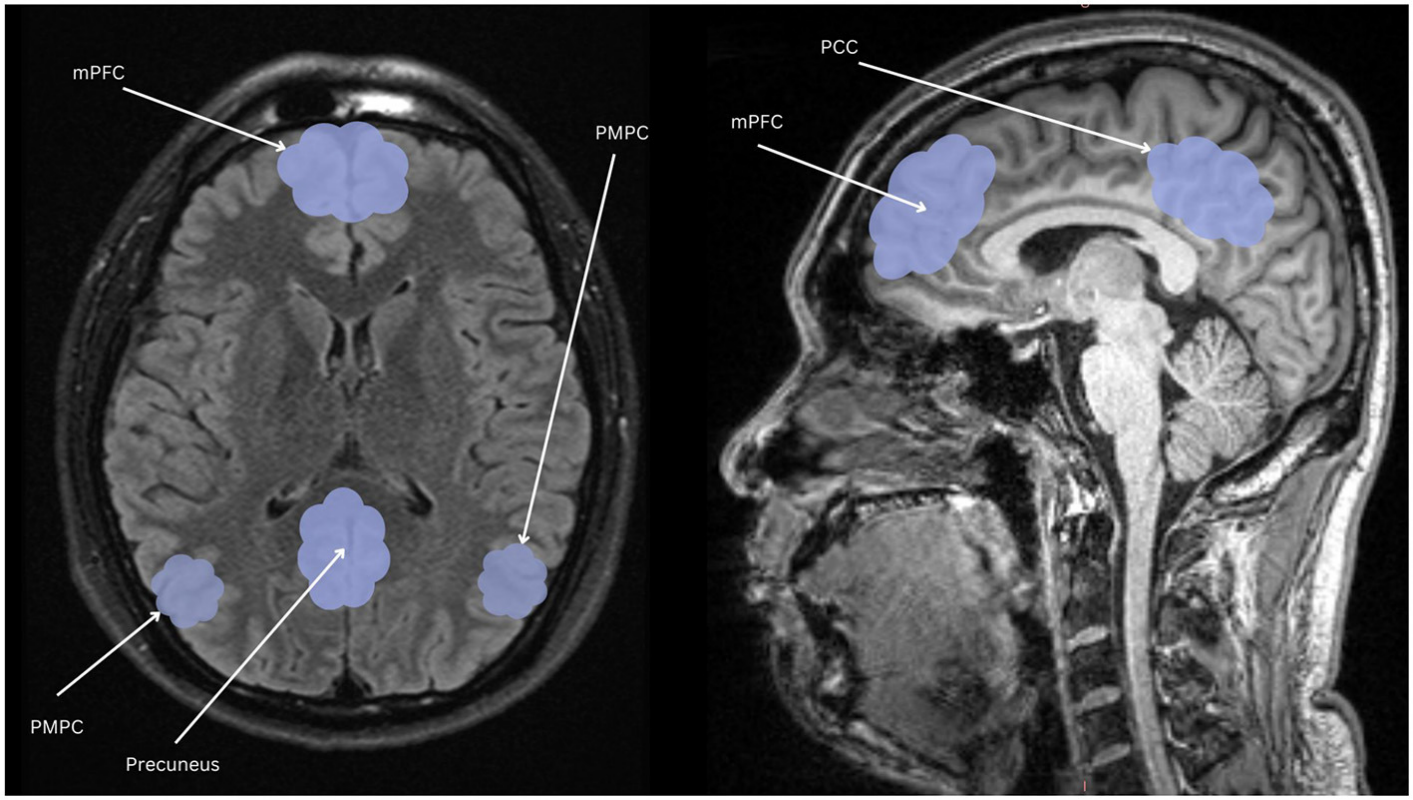
Nodes of the DMN highlighted in light blue: mPFC, the medial prefrontal cortex; PCC, posterior cingulate cortex; PMPC, precuneus, and posterior medial parietal cortex.

The symptomatic presentation of PTSD is partially attributed to abnormal brain functioning associated with resting state activity, now known as the default mode network (DMN). While resting state activity was noted during the rise of EEG use in studies, it was centrally used as a control and even considered noise by much of the scientific community (Chan, 2021). This idea was entirely reversed by two critical studies (Shulman et al., 1997; Raichle et al., 2001) which formally identified the DMN as a network of regions in the brain that activate simultaneously in support of intrapersonal cognition during resting states. Substantial studies now support the DMN's role in subserving baseline cognitive activities, such as self-referential processing, social cognition, autobiographical recall, and prospection (Menon, 2023; Chan, 2016). Neuroanatomically, popular nodes include the medial prefrontal cortex, posterior cingulate cortex, bilateral inferior parietal, posterior temporal regions, bilateral temporoparietal junction, precuneus and the parahippocampal cortex (Menon, 2023; Chan, 2016; Christoff et al., 2016; Andrews-Hanna et al., 2014). More recently, subcortical regions have also been added including the anterior and mediodorsal thalamic nuclei and the basal forebrain (Alves et al., 2019). Given robust evidence for DMN disruption in neurocognitive diseases and disorders^1^ (Etkin and Wager, 2007), it is likely a significant percentage of trauma-exposed individuals without PTSD will have alterations in neuropsychological functioning. Clinically, a high proportion of patients are symptomatically treated for covert traumatic symptoms that do not meet the threshold for the diagnosis of PTSD. Currently, few studies focus on DMN function in trauma-exposed individuals without PTSD, which could uncover unique factors pertinent to early prevention. Extant research suggests that the DMN is one of the first networks affected after a traumatic event, thus conferring value to its potential predictive, diagnostic, and prognostic capacity (Lanius et al., 2010; Zhou et al., 2012). Once established, clinical correlation of biopsychological determinants of DMN dysfunction and traumatic syndromes can offer novel insight into objective indicators of the development and severity of trauma-induced pathophysiology. Ongoing identification of these correlates is thus essential to expanding our understanding of trauma-related sequelae and enhancing evidence-based practices. Table 1 identifies current regions associated to the DMN, followed by Figure 1 providing a visual depiction.

**Table 1:** Subdivisions of the DMN with associated regions and functions.

**Default network subsystems (Christoff et al., 2016)**	**Associated regions**	**Functions**
DN_CORE_	• Anterior mPFC • Posterior cingulate cortex • Posterior inferior parietal lobule	• Self-referential processing • Episodic memory • Future planning • Mind wandering
DN_MTL_	• Hippocampal formation • Parahippocampal cortex	• Memory and learning • Autobiographical recall • Prospection • Fear conditioning • Perceptual processing
DN_SUB3_	• Dorsomedial prefrontal cortex • Lateral temporal cortex extending into the temporopolar cortex	• Social cognition • Emotion processing • Theory of mind

### PTSD, trauma, and functional imaging

Neuroimaging studies have identified DMN characteristics distinctive of PTSD psychopathology. Principal cognitive functions linked to DMN activity patterns, including autobiographical recall, prospection (Spreng et al., 2009), self-referential processing (Lanius et al., 2011), and social cognition (Mars et al., 2012). While these fundamental DMN processes are normally moderated by the salience network (SN) and suppressed by the central executive network (CEN), they are also susceptible to systematic reconfiguration. One distinguishing feature is “amygdala hijack,” which allows reflexive emotions to override regulatory signals and mediates fear conditioning. Perpetuation of amygdala hyperactivity inhibits higher-order input and leads to increased arousal, avoidance, and intrusion symptomatology. This is supported by Zhu et al.'s (2018) study demonstrating decreased functional connectivity between the amygdala and the left parahippocampus in patients with PTSD compared to trauma-exposed controls. Though amplified amygdala-hippocampal activation is seen during the induction of negative affective states (Brohawn et al., 2010), reduced connectivity in neutral states appears to relate to PTSD deficits in properly contextualizing threat and safety signals. Sripada et al. (2012) also observed hyperconnectivity of the amygdala and right insula in veterans with PTSD, likely reflecting the role of insula responsivity in emotion dysregulation and intensity of hyperarousal and re-experiencing symptoms. Finally, an fMRI analysis by DiGangi et al. (2016) found that combat veterans showed weaker resting-state mPFC connectivity to the precuneus and right superior parietal lobule, which may contribute to negative self-evaluation and reduced insight and judgment. Though traumatic events—with or without ensuing PTSD—may induce DMN changes, the consequences are not invariably consistent and may be contingent on heritable or acquired traits, adding further heterogeneity to cases to this area of study.

Regarding trauma-exposed individuals explicitly without PTSD, Kennis et al. (2015) identified increased functional connectivity between the ventral anterior cingulate cortex (DMN) and right middle frontal gyrus (CEN) when compared to both PTSD and healthy control groups. Zhang et al. (2018) similarly showed increased functional connectivity in the superior frontal gyrus in trauma-exposed subjects compared to healthy controls. These intergroup disparities may be due to resilience factors that maintain effective executive functioning, and at some level buffer the harmful effects of the traumatic experiences. Thus, consistent patterns of network connectivity variations are noteworthy, as they likely indicate dynamic mechanisms underlying modified stress responses and diverse trauma manifestations. Network adaptations that foster protective traits are particularly crucial in the context of childhood trauma and stress vulnerability. In subjects with early trauma exposure, executive dysfunction severity appears to correlate with reduced DMN connectivity. Altered precuneus activity of otherwise healthy adults with childhood trauma exposure is particularly notable, as it is comparable with DMN dysfunction seen in combat-related PTSD patients (Lu et al., 2017). DMN changes associated with early life stress occur during a critical brain maturation period and are disruptive to normal developmental processes. These changes are enabled by neuroplasticity, which is essential to, as well as dependent on, learning and memory. While experience-dependent neuroplasticity is exceptionally prominent in childhood, its structural sensitivity grants opportunities for optimally timed intervention and advantageous neural reorganization. Considered collectively, clinical markers of DMN function may further focus management approaches and mitigate the ramifications of trauma.

### The impact of trauma on mental time travel: autobiographical recall and prospection

Autobiographical memories (AMs) are self-referential, long-term memories comprised of both episodic and semantic details that can be retrieved voluntarily or involuntarily. Correspondingly, autobiographical recall is the ability to access and utilize firsthand narratives of past experiences to facilitate decision-making and learning. This involves the integration of several cognitive processes, including self-referential processing (SRP) and theory of mind (ToM). According to Spreng et al. (2009), reconstructing scenes from memory requires the projection of oneself into the past via retrieval of stored memories, including emotional, sensory, spatial, and temporal information. Naturally, this serves as an anchor for identity formation and stability of self-concept. Expectedly, individuals with trauma exposure often struggle with memory-related functions, with AMs constituting one of the most disrupted memory systems. Defective encoding can lead to memory fragmentation, improper contextualization, and flawed perceptual interpretations. These limitations likely contribute to the overgeneralized, negative cognitions associated with PTSD.

Alterations in memory processes are commonly seen in individuals with PTSD, who display overgeneral autobiographical memory (OGM) bias during memory recall (Ono et al., 2016). OGM is a phenomenon whereby individuals recall categorical memory representations with reduced specificity and vividness as opposed to discrete autobiographical details. For instance, when asked to recall a happy event, a person exhibiting OGM may say, “When I traveled for the holidays,” rather than a specific incident, such as “When I celebrated my birthday last year with my family and friends.” Though the effect of valence on cue response can be variable, OGM has been implicated as a pre-disposing risk factor for symptomatic trauma disorders (Moore and Zoellner, 2007). Comparatively, Schönfeld and Ehlers (2017) found that trauma-related memories tend to be experienced as more specific, vivid, and associated with a sense of “nowness,” which may incite intrusion, arousal, and dissociative symptoms in those with Post-traumatic Stress. Such disruptions often impact attention and concentration and contribute to the heightened arousal that are often reported in individuals with PTSD. Thus, memory specificity could be deemed as disruptive (e.g., misguided reactivity, poor decision-making), and overgenerality regarded as a coping mechanism (e.g., suppression, avoidance). The over-general retrieval of non-traumatic memories is also associated with feelings of discontinuity between past- and present-self, causing a sensory-emotional detachment that destabilizes the sense of self, social connectedness, and temporal perception. These conceptual distortions limit goal- and future-oriented thinking, which rely on the quality of contextual information during episodic memory recall.

Along with memory utilization, the DMN is also linked to mind wandering and imagining of future events, an ability known as prospection. Mental simulation of various potential futures is adaptive, as it enables advanced planning to maximize the probability of achieving a goal (Spreng et al., 2009). Analogous to AM, trauma exposure can result in reduced specificity and vividness of prospective simulations during mental time travel (MTT). Kleim et al. (2014) found that compared to healthy controls, trauma survivors diagnosed with PTSD were able to imagine fewer specific future events in response to positive cues. These differences in specificity remained significant after controlling for trauma-relatedness of the memories, ruling out the explanation that the results were due to biased memory access. Brown et al.'s (2013) examination of combat veterans suggests that trauma-exposed individuals with PTSD show similar AM and MTT deficits when generating past and future events. These findings are consistent with the notion that autobiographical memory and prospection rely on similar cognitive mechanisms (D'Argembeau, 2021). However, there may be a discrepancy in image vividness and specificity between trauma-exposed individuals with and without PTSD. Brown et al. (2013) investigated these differences and found that those with PTSD generated more external (i.e., non-episodic, general) than internal (i.e., episodic, specific) details when recalling the past or imagining the future, with reduced internal details correlating with greater symptom severity.

Below is a table from an extensive review by Chan (2016) summarizing findings characteristic of a traumatized resting state. The conclusion of this review indicated a neurodynamic imbalance between the DMN, CEN and SN. More specifically, intrusions of the DMN during goal-directed tasks, which normally activate the CEN (which in healthy cases suppresses the DMN) and intrusions upon the DMN from the Salience Network. These may reflect intrusive experiences related to traumatic autobiographical memories during externally focused attention toward tasks, and instability of salience detection which may relate to hypervigilance. Table 2 summarizes findings related to how PTSD impacts the Default Mode Network.

**Table 2:** Neuropsychological profile of DMN dysfunction: traumatized resting state.

**Neural functioning**	**Description**
• Reduced connectivity of mPFC and limbic regions. Hypoactivity in mPFC and hyperactivity in the amygdala. • Increased connectivity and activation of PCC with the right amygdala	• Increased emotional reactivity, aberrant self-referential processing. Reduced ability for top-down regulation. • Threat, survival, negative biased processing in the evaluation of self, environment and emotion mediated memory.
Decreased connectivity between PCC with precuneus, bilateral parietal cortex, and mPFC	Inaccurate (biased) and decreased capacity for evaluation of self, prospection, social cognition, and moral sensitivity.
• Reduced left hippocampal functioning • Increase in the right hippocampus	• Reduced verbal, and autobiographical recall (context-dependent) • Increased visual memory (priming for action), increased processing of negative memory
Decreased activity in precuneus, mPFC, and right superior parietal lobule	Reduced capacity for self-reflection and biased prospection. Differences in mental imagery.
Reduced connectivity with ACC with mPFC and medial temporal gyrus	Altered attentional control, error monitoring, decision-making and emotional pain perception
Increased Insula activity and connectivity with SN (amygdala)	Alterations in interoception (i.e., exaggerated bodily response) and attentional activity
Reduced insula connectivity PCC	Interoceptive activity is less connected to introspective activity, and emotion-mediated memory.
Dissociated left and right amygdala	Imbalances in emotion processing, and difficulties regulating its cognitive, emotional, and autonomic expressions.

Table is reprinted and elaborated on with permission from Chan (2016).

Regarding trauma-exposed individuals explicitly without PTSD, Kennis et al. (2015) identified increased functional connectivity between the ventral anterior cingulate cortex (vACC, part of the DMN) and right middle frontal gyrus (CEN) when compared to both PTSD and healthy control groups. Zhang et al. (2018) similarly showed increased functional connectivity in the superior frontal gyrus in trauma-exposed subjects compared to healthy controls. Put together, such differences between groups may suggest protective factors that supports the regulation of mood, cognition and the autonomic nervous system. Thus, consistent patterns of network connectivity variations are noteworthy, as they likely indicate dynamic mechanisms underlying modified stress responses and diverse trauma manifestations. Network adaptations that foster protective traits are particularly crucial in the context of childhood trauma and stress vulnerability. In subjects with early trauma exposure, executive dysfunction severity appears to correlate with reduced DMN connectivity. Altered precuneus activity of otherwise healthy adults with childhood trauma exposure is particularly notable, as it is comparable with DMN dysfunction seen in combat-related PTSD patients (Lu et al., 2017). DMN changes associated with early life stress occur during a critical brain maturation period and are disruptive to normal developmental processes. These changes are enabled by neuroplasticity, which is essential to, as well as dependent on, learning and memory. While experience-dependent neuroplasticity is exceptionally prominent in childhood, its structural sensitivity grants opportunities for optimally timed intervention and advantageous neural reorganization. Considered collectively, clinical markers of DMN-related functions may further focus management approaches and mitigate the ramifications of trauma.

### The impact of trauma on self-referential functions

Self-Referential Processing (SRP), the process of interpreting stimuli as they relate to oneself, correlates with DN_CORE_ subsystem functioning. Northoff et al. (2006) characterize self-referential processing as an experience that is “implicit, subjective and phenomenal (p. 441).” This is presupposed by pre-reflective consciousness and the faculty of self-perception, which underlies higher-order expressions of awareness. Current models consider the self as a baseline that serves the function of a reference point for inputs from the surrounding environment. Large scale meta-analysis from Scalabrini et al. (2024) led to the proposal of three hierarchically nested levels: (1) the interoceptive which corresponds to the awareness of internal signals from our body, the extero-proprioceptive, that relates to external sensory inputs, and (2) the mental, which refers to psychological faculties such as self-referential processing. They also encountered that relational based traumas (e.g., abuse) incurred differential effects on the self than non-relational based trauma (e.g., natural disaster), with the former impacting the interoceptive and extero-proprioceptive levels, and the latter the mental level. Of note, the mental level corresponds to default mode activity, which incorporates certain areas from the previous two levels (hence nested) such as the bilateral insula and thalamus from the interoceptive level and the bilateral insula, and temporoparietal junction from the exteroceptive. This may suggest that individuals who have experienced a non-relational trauma may have the capacity to recruit higher order psychological strategies to emotionally regulate, whereas those with more intimate traumatic experiences from relationships, may experience dysregulation at a more bodily level, unable to more fully access symbolic means of regulation. Importantly, all three levels are connected by the insula, which is known to be related to functions such as integrating internal somatic and emotional signals with external somatic information to help coordinate decision making (Uddin et al., 2017). As such, in combination with the DMNs reflective capacities, it can be understood as being essential for facilitating a comprehensive experience of the embodied self. DMN studies have investigated SRP via examination of subjects' responses to statements describing their own personality, attitudes, or preferences. These context cues stimulate activity in DMN core areas, including the medial prefrontal cortex, posterior cingulate cortex, precuneus, and other regions (van Buuren et al., 2012). SRP enables the use of both internal and external sources of self-appraisal to extrapolate inferences, whereby self-relevant information is encoded and applied to self-concept integration. Correspondingly, social insights are abstracted via mental representation and simulation, as self-reflective capacities are also used to model the cognitive states of others (Lanius et al., 2020). This intuitive function involves the integration of self- and other-referential processing. When intact, these reciprocal processes ensure effective intra- and interpersonal functioning and promote appropriate behavior. Conversely, trauma-related impairment of these processes limits adaptive functioning and decreases prosocial behavior germane to healthy psychosocial development. Stress and trauma, especially repeated often, intensify the relationship between self- and trauma-processing and can increase the severity of PTSD symptomatology (Lanius et al., 2020).

Traumatic experiences that diminish SRP capacities cause preferential retrieval of learned fear paradigms and lead to maladaptive thinking patterns, such as cognitive distortions and negative self-talk. The corresponding irrational beliefs can become embedded in one's self-concept and perpetuate psychopathological conditions. Negative thinking also mediates the direct neuroendocrine effects of stress, which correlate with amygdala hypersensitivity and alteration of the hypothalamic-pituitary-adrenal (HPA) axis feedback loop. Studies on adults with a history of childhood abuse demonstrate increased cortisol levels and sympathetic overactivation in response to perceived psychosocial stressors. When cortisol signaling is frequent or faulty, chronic HPA axis dysregulation develops, causing nervous system imbalances with rampant top-down and bottom-up signals producing persistent somatic and physiologic disturbances. This neurobiological response also prompts DMN structural connectivity changes to maintain heightened attentiveness and alertness as an anticipatory strategy to confront perceived threats, thus reinforcing a dysregulated stress response with detrimental health implications. These findings indicate that stress exposure, especially adverse childhood events, confers commensurate fragility to fundamental DMN processes. Though this implies that early life stressors can create a predisposition toward PTSD (Lanius et al., 2020), trauma-related effects on neural reorganization are moderated by genetic variations in cortisol response, which may contribute to stress resilience and relative sparing of SRP functions.

### The impact of trauma on social cognition

Social cognition is closely associated with SRP and is comprised of several cognitive domains, including the theory of mind, emotion processing, social perception, and attribution style (Pinkham et al., 2014). Among these capacities, studies commonly implicate DMN connectivity as a crucial component of the Theory of Mind (ToM) (Li et al., 2014). ToM is the ability to recognize the subjective mind of others as analogous to one's own, infer their mental state, and attribute meaning (e.g., motives, intentions, and opinions) (Pinkham et al., 2014). This ability is fundamental to developmental learning and social competence, which determine the quality of our social interactions and relationships. Studies have shown that individuals with PTSD demonstrate deficiencies in social cognition, including ToM, emotion recognition, and social inference (Couette et al., 2020; Stevens and Jovanovic, 2018). These impairments can significantly affect intersubjective experiences and social reasoning skills, resulting in misinterpretation of social cues and interpersonal difficulties that lead to withdrawal and self-isolative behaviors seen in those with PTSD (Couette et al., 2020). Trauma-exposed individuals without PTSD also display deficits in social cognition, as evidenced by impaired emotion recognition compared to healthy controls (Stevens and Jovanovic, 2018), raising the notion that trauma exposure alone can impact social function.

Lee et al. (2022) found that childhood trauma moderates the relationship between ToM and hair cortisol concentration (HCC) in healthy young adults. This suggests that ToM deficits can cause high levels of social stress, which are intensified by increased stress sensitivity in the setting of historical childhood trauma. Even with early trauma exposure, normal HCC is seen in individuals without psychiatric illness and may indicate resilient characteristics. Specific modalities of childhood trauma can also impact ToM abilities differently. Childhood emotional abuse was associated with reduced theory of mind accuracy in depressed individuals, while physical abuse was associated with reduced accuracy in non-depressed individuals (Rnic et al., 2018). Interestingly, those with a history of neglect showed enhanced theory of mind decoding accuracy, possibly suggestive of an adaptive coping strategy and manner of acclimating to environmental lack of control. In contrast, Simon et al. (2019) found that childhood adversity, particularly emotional abuse and neglect, was associated with inferior ToM abilities. The disparity among findings suggests that the type of childhood adversity, along with biopsychosocial factors, likely have differential effects on social cognition, and these effects may be further moderated by other variables.

It is imperative to note that DMN activities relating to self, social, and memory processes are intricately interdependent. The inherent overlapping of these cognitive domains and reliance on DMN integrity reinforces trauma's plausible magnitude of impact. Conversely, their interdependent mechanisms may also contain potential foci of high-yield clinical value. A primary step to improve trauma-exposed patient outcomes is identifying high-risk individuals expected to benefit from prompt, targeted intervention. Currently, the predictive validity of standardized screening tools varies broadly, underscoring the utility of supplementary DMN data for detecting less overt neurocognitive manifestations and approximating clinical trajectories. The goal of our study is to objectively measure DMN functions and their corresponding structural differences in individuals who suffered a qualifying traumatic experience that did not result in diagnosable PTSD. In this study, we hypothesized that cognitive functions associated with the DMN would be mixed, with some enhanced, diminished, or unchanged. We anticipated a negative bias influencing the self-referential effect to be evident given prior findings, including lower scores on social cognitive measures and reduced quality of memory recall and prospection. We further hypothesized a reduction in mind wandering and volumetric DMN changes in trauma-exposed subjects relative to controls. Protective factors cited from previous studies include greater volumes of the thalamus and rostral middle frontal gyrus, greater volume of the right temporal lobe (Nilsen et al., 2016), unchanged hippocampal subfield volumes (Postel et al., 2021; Koch et al., 2021), and increased mPFC thickness (Jeong et al., 2021).

### The neuropsychology of trauma resilience

Resilience to trauma and stress has been associated with specific patterns of brain activity, structural integrity, and functional connectivity across neural regions, alongside broader cultural and coping factors. Higher resilience, marked by increased connectivity between the medial Prefrontal Cortex (mPFC) and other brain regions, was linked to reduced cross-network connectivity between the Default Mode Network (DMN) and Salience Network (SN), suggesting a protective role against PTSD symptoms (Brunetti et al., 2017). Similarly, greater cortical thickness in specific brain regions associated with emotional semantic memory processing indicated potential resilience against PTSD development (Nilsen et al., 2016). Additionally, increased hippocampal activation during fear conditioning and subsequent contextual fear extinction was associated with higher resilience and lower PTSD symptoms post-trauma, highlighting the significance of hippocampal involvement in resilience (van Rooij et al., 2021). Investigations into social support, intrapersonal resilience, and coping mechanisms have shown mixed associations with PTSD severity (Rakesh et al., 2021; Besser et al., 2014; Lee, 2019). Research into biologically based mechanisms such as behavioral synchrony has identified specific pathways contributing to resilience, particularly in children exposed to trauma (Motsan et al., 2020). In terms of cultural contributions to resilience, significant differences in resilience scores among diverse ethnic backgrounds have been noted, emphasizing the importance of factors such as religious coping and ethnic pride (Raghavan and Sandanapitchai, 2019).

Larger gray matter volumes in the rostral middle frontal gyrus and thalamus and effective emotion reappraisal strategies are linked to better outcomes after early-life stress (Zilcha-Mano et al., 2022; Park et al., 2022). The central executive network (CEN), including the dorsolateral PFC (DLPFC), protects against cardiometabolic risks in youth exposed to environmental stressors (Miller et al., 2018). Enhanced cross-network connectivity between the medial PFC, Default Mode Network (DMN), and Salience Network (SN) reduces PTSD symptoms, while mixed findings on social support and intrapersonal resilience suggest variability in coping mechanisms (Brunetti et al., 2017; Rakesh et al., 2021; Besser et al., 2014; Lee, 2019). Cultural contributions may play a role, with significant differences in resilience scores across ethnic groups emphasizing factors like religious coping and ethnic pride (Raghavan and Sandanapitchai, 2019).

Cognitive factors also play a role in trauma resiliency. Ben-Zion et al. (2018) conducted two independent studies examining the role of neurocognitive factors in trauma survivors in relation to PTSD symptoms and resiliency. In the first study, individuals with better cognitive flexibility at 1-month post trauma showed significantly less severe PTSD symptoms after 13 months. In the second study, trauma-exposed individuals who underwent neurocognitive training showed more improvement in both cognitive flexibility and decreased PTSD symptoms 6-months post-trauma compared with controls. Furthermore, individuals who demonstrated the largest improvements in cognitive flexibility showed greater clinical improvement. Taken together, these results suggest that cognitive flexibility is a significant predictor of PTSD symptom severity and that neurocognitive interventions post-trauma can lead to less severe clinical symptomology. These findings are in line with previous research that has linked poor cognitive flexibility with an increase in PTSD symptom severity (Scott et al., 2015), and suggest that cognitive flexibility is a significant marker of trauma resiliency that can provide a buffer against the development of more severe PTSD symptomology. Greater cognitive flexibility enables trauma-exposed individuals to differentiate between threat-related and neutral situations, allowing for improved adaptability to environmental circumstances.

### Pre-trauma neural features

Longitudinal studies have captured pre-trauma structural and functional neural features that predict changes in later brain function and adaptive natural recovery from stress-related symptoms after trauma. These findings shed light on neural resilience and offer insights into how the brain changes and recovers after exposure to psychological stress.

The amygdala constitutes a key structure involved in pre-trauma resilience. Individuals with less amygdala activity measured before trauma exposure were found to be more resilient and reported fewer PTSD symptoms post-exposure, in children studied before a terrorist event (McLaughlin et al., 2014) and young adults prior to military deployment (Admon et al., 2009, 2012; Lin et al., 2017), with effects generalizing beyond PTSD to more general stress-related symptoms. Individuals with less amygdala reactivity may also demonstrate less sympathetic output when countering new threats (Samuels and Szabadi, 2008). Further supporting this, a longitudinal study of adolescents with a history of childhood maltreatment found that lesser amygdala threat reactivity uniquely predicted resilience, as reflected by lower internalizing symptoms, independently of life stressors or socioeconomic factors (Gerin et al., 2019). Biologically-based mechanisms such as behavioral synchrony identify pathways contributing to resilience, particularly in children exposed to trauma (Motsan et al., 2020).

The prefrontal cortex (PFC) plays a central role in emotional regulation and resilience, with increased ventromedial PFC (vmPFC) responsivity to aversive stimuli linked to active coping strategies and reduced maladaptive behaviors, such as emotional eating and alcohol consumption (Sinha et al., 2016; Fonzo et al., 2017). The vmPFC is also an important contributor to adaptive threat responses, implementing top-down control over fear-related regions like the amygdala, ultimately helping to modulate threat responses adaptively (Fitzgerald et al., 2018). Overall, greater vmPFC function, volume, and connectivity have been linked with greater stress resilience (Roeckner et al., 2021). Neurofeedback training enhancing PFC regulation of the amygdala improves emotional control (Koush et al., 2017). In a military predeployment sample, EEG-based amygdala downregulation training decreased amygdala BOLD signal and increased amygdala-mPFC connectivity, improving experimental emotional regulation indices (Keynan et al., 2018).

The hippocampus is another key structure, with greater hippocampal volume and greater bilateral hippocampal activity both predictive of resilience to PTSD symptoms measured post-exposure (Pitman et al., 2006; McLaughlin et al., 2014; Rubin et al., 2016). Enhanced hippocampal activation during fear conditioning and extinction, as well as structural covariance networks involving the hippocampus and PFC, are linked to resilience in trauma-exposed populations (van Rooij et al., 2016, 2021; Sun et al., 2018, 2019).

The salience network, composed primarily of the insula and ACC and involved in the monitoring of affective environmental cues, constitutes another center key in trauma resilience. The anterior cingulate cortex (ACC), particularly the dorsal ACC (dACC), also contributes to resilience. Increased dACC resting metabolism is associated with PTSD symptom severity, while lower amygdala and hippocampal resting metabolism in trauma-exposed individuals without PTSD may signify a resilience factor (Marin et al., 2016; Kaldewaij et al., 2021; Brosch et al., 2021). Greater cortical thickness in regions related to emotional semantic memory processing further supports resilience to PTSD development (Nilsen et al., 2016). Less resting state dACC and mid-cingulate activity is associated with resilience to the development of PTSD (Shin et al., 2009), as well as less responsivity of the dACC during a cognitive interfering task (Shin et al., 2011). Taken together, less functional activity in the dACC pre-trauma may constitute a protector factor.

### Peri-trauma and post-trauma neural features

The structure and function of areas involved in threat inhibition, threat response, and salience detection, including the amygdala, insula, dACC, dmPFC, hippocampus, and vmPFC, are consistently implicated in peri- and post-trauma resilience. Amygdala reactivity to negative emotional stimuli negatively correlates with resilience as measured by fewer PTSD symptoms months after the trauma (Stevens et al., 2017). However, individual differences in amygdala structure vis-a-vis the development of peri/post-trauma resilience is not as clear. The dACC and insula, two other areas that also overlap between the threat and salience network, are associated with resilience. A pattern of larger insula, dACC, and rACC volume measured shortly after trauma is predictive of fewer future trauma symptoms after head injury and motor vehicle accident (Stein et al., 2021; Hu et al., 2018), which may be related to greater adaptive regulation of emotional responses to threat stimuli. The picture is more complicated in terms of functional activity of the threat and salience network. Anterior insula and dACC activity during an affective task requiring effortful attention allocation has been shown to correlate with PTSD symptom improvement (White et al., 2018). However, PTSD symptom improvement was also negatively correlated with dmPFC engagement during a less effortful emotional task in the 2 weeks after a motor vehicle collision and with insula engagement 3 months after. Furthermore, dmPFC activation was correlated with an increase in PTSD severity over this time (Wang et al., 2016). The threat and salience network's contribution to trauma resilience may depend on its role in task demands, including attention allocation and the emotional context and nature of the trauma. Increases in vmPFC volume and activity is also important for later resilience. Trauma survivors with greater rACC and vmPFC volume and surface area collected peri-trauma were less likely to develop PTSD symptoms in the 3–12 months following the trauma (Stein et al., 2021). Diffuse tensor imaging studies also show that larger fractional anisotropy and less mean diffusivity in the vmPFC predicted resilience 2 days following a traumatic event, and larger vmPFC fractional anisotropy predicted lower PTSD severity in the months following the trauma (Sun et al., 2013). Overall, it appears that greater vmPFC volume, fractional anisotropy, and engagement in the context of threat predict lower PTSD severity post-trauma, and may reflect the role this area plays in inhibiting fearful responses. Hippocampal structural integrity and activation are also predictive of resilience, with larger hippocampal volumes early post-trauma correlating with greater resilience (Xie et al., 2018). Increased hippocampal activity during response inhibition 1-month after a trauma is predictive of PTSD resilience, with lower symptom severity up to 6 months post-trauma (van Rooij et al., 2018). Activation of the hippocampus during fear conditioning is also positively correlated with resilience at 2 months post-trauma (van Rooij et al., 2021). Traumatic events that result in smaller hippocampal volume may lead to impaired functionality, or smaller hippocampal volume and/or reduced functionality may constitute a vulnerability factor. Overall, hippocampal and vmPFC modulation of amygdala activity early post-trauma supports resilience and recovery, and less amygdala reactivity, greater hippocampal activity, greater vmPFC activity and volume, and greater functional and structural connectivity between these regions and the amygdala early post-trauma correlate with improvements in symptoms and better PTSD recovery.

The DMN has consistently been implicated in resilience across early post-trauma studies. Greater FA across nodes of the network, greater cortical thickness, GMV, and surface area within DMN nodes all positively predict resilience in the weeks and months following trauma (Sun et al., 2013; Busso et al., 2017; Cwik et al., 2019, Heyn and Herringa, 2019). Once again, the picture is quite mixed, as functional studies have found that early post-trauma DMN engagement via the PCC and threat network connectivity can have a negative impact on resilience, mainly within the first year after a trauma (Roeckner et al., 2021). However, while DMN engagement and threat-network connectivity predict acture trauma-related symptoms, they may lead to improvements in symptoms and resilience in the long-term. For example, reactivity in the DMN to trauma-related pictures 2 months after a trauma was negatively correlated with early PTSD resilience, but 2 years later, the same activity was correlated with better resilience (Ke et al., 2016). It appears that the DMN plays an important role in treatment and recovery, but functional connectivity within and with the DMN may fluctuate over time, and these fluctuations impact both early and later resilience.

The attention network is another key network involved in trauma resilience, both in the acute stage after trauma and in long-term recovery. Inferior parietal lobule activity both early post-trauma in response to trauma-related images and in response to emotional stimuli 2–12 months post trauma predicted resilience and symptom reduction (Kaldewaij et al., 2021; White et al., 2018). Larger volume and greater FA of the superior longitudinal fasciculus early posttrauma appeared protective against the future development of PTSD after military combat and motor vehicle collisions. The attention network may be a particularly beneficial target for facilitating recovery given that it is consistently implicated in trauma resilience in multiple types of traumatic experiences and different cohorts. The cognitive control network is also implicated in resilience, with changes in the structure and function of the dlPFC particularly predict resilience (Lyoo et al., 2011). The dlPFC may play a role in the regulation of emotional arousal responses during the early recovery period, as dlPFC connectivity with the bilateral amygdala and brainstem 2 weeks after a trauma predicts subsequent resilience to PTSD and depressive symptoms in the months following (Heyn et al., 2019; Harnett et al., 2021). These results suggest that the dlPFC may constitute an appropriate target for early intervention (neuromodulation, etc.).

Some sensory areas show associations with resilience over time, such as the occipital cortex and fusiform gyrus, which are involved in vision, and the precentral cortex, which is involved in the regulation and relaying of sensory inputs. Visual activity within the first year after a trauma seems to negatively correlate with resistance (Roeckner et al., 2021). Increased occipital centrality during rest-state 3 weeks after a sexual assault was able to predict PTSD diagnosis 6 months after the trauma (Quidé et al., 2021). Peritraumatic dissociation, which constitutes a known risk factor for later chronic PTSD, depression, and chronic pain (Beaudoin et al., 2021; Kessler et al., 2020), correlates with activity in the right ventral visual pathway, fusiform, lingual, and parahippocampal gyrus during a trauma script task 2–4 months later (Daniels et al., 2012). This results suggest a negative correlation between visual circuit activation and resilience. Overall, it appears that activation of sensory areas negatively correlates with both early resilience and later recovery in the peri-trauma stage. Table 3 summarizes pre-trauma and peri-trauma factors.

**Table 3:** Summary of pre-trauma and peri-trauma factors.

	**Neural features**	**References**
**Pre-trauma factors predicting resilience**		
• Threat-inhibition (positive correlation) • Cognitive control (positive correlation) • Threat-response (negative correlation) • Salience networks (negative correlation)	• Larger vmPFC and hippocampal volume • Increased activation of vmPFC, hippocampus, aPFC • Decreased activation of amygdala, dACC, and LC • Greater vmpFC and hippocampal coupling	Roeckner et al., 2021; Gilam et al., 2017; Kaldewaij et al., 2021; Admon et al., 2009; Stevens et al., 2017; Grueschow et al., 2021
**Peri-trauma factors predicting early resilience**		
• Threat-inhibition (positive correlation) • Cognitive control (positive correlation) • Attention (positive correlation) • Salience (positive correlation) • Reward (positive correlation) • Sensory (negative correlation) • Default mode (negative correlation)	• Conserved or larger structural features in hippocampus, parahippocampus, vmPFC, sgACC, dlPFC, temporal lobe • Greater rACC surface area volume • Decreased amygdala functional activity • Increased hippocampal/parahippocampal functional activity • Decreased DMN activity	Roeckner et al., 2021; Dickie et al., 2011; Stein et al., 2021; Hu et al., 2018; Admon et al., 2009; Quidé et al., 2021; Ke et al., 2016
**Peri-trauma factors predicting later recovery**		
• Threat-inhibition (positive correlation) • Cognitive control (positive correlation) • Attention (positive correlation) • Salience (positive correlation) • Reward (positive correlation) • Default mode (positive correlation) • Threat-response (negative correlation)	• Conserved or larger structural features in hippocampus, parahippocampus, vmPFC, • Increased DMN activity • Decreased amygdala functional activity • Increased hippocampal/parahippocampal functional activity • Structural increases in OFC	Roeckner et al., 2021; van Rooij et al., 2021; Dickie et al., 2011; Harnett et al., 2021; Admon et al., 2009; Ke et al., 2016

## Study purpose and hypothesis

The purpose of this systemic exploratory study is to examine how DMN structure and function relates to trauma exposure. This is approached from a neuropsychological perspective. As indicated in the preceding review, previous research has correlated DMN functioning with specific cognitive tasks. As such, DMN functioning was examined through relevant neurocognitive measures. In addition, much of the imaging work conducted has been functional in nature, and not structural. With function being addressed by neuropsychological assessments, and with greater mystery surrounding potential structural differences underlying protective factors, the collective decision was to examine the DMN through structural imaging.

We hypothesize that trauma exposure would likely be related to significant differences in function and to some extent structure. As an attempt to pick up on more subtle alterations, we also incorporated an analysis of hippocampal subfields. The hippocampus has previously been identified as an important region related to resilience to PTSD (van Rooij et al., 2021) and it has been proposed that reduced hippocampal volume may be a predisposing factor, rather than a consequence of PTSD (Rubin et al., 2016). As such, a deep look into the hippocampus may provide finer grained observations that supports or does not support this theory.

We hypothesize that similar to previous studies listed in this review, there will be significant reductions in self-referential processing, social cognitive processing, autobiographical recall, prospection, and increased mind-wandering which may be reflected in structures such as the medial prefrontal cortex, posterior cingulate cortex, hippocampus and the surrounding temporoparietal regions.

## Materials and methods

### Participants

A total of 27 participants were recruited and allocated into two groups, including 16 trauma-exposed (TE) without PTSD and 11 in the non-trauma (NT) control group. There were 13 male and 14 female subjects, ranging in age from 18 to 59 years. The control group was comprised of five males and six females; TE group has eight males and eight females.

Recruited subjects were evaluated twice, once for neuropsychological assessment and once for neuroimaging, should they have agreed to and were determined eligible to participate in MRI. Imaging was completed for a total of 15 participants (eight males and seven females), which included six control (three males, three females) and nine TE (five males, four females) subjects. One TE individual was excluded due to artifacts with low-resolution scans. All subjects were given the Mini International Neuropsychiatric Interview, BDI-II, and BAI to ensure they were not suffering from significant mood disturbances. TE groups were additionally given the Life Events Checklist with subsequent administration of the Clinician-Administered PTSD Scale (CAPS-5) to rule out a PTSD diagnosis. Individuals were also native or fluent English speakers. The measures being used have not yet been translated to any other languages, and the battery included two novel measures, which were designed for English speakers. Subjects indicating a suboptimal performance (according to an effort measure—Dot Counting) were also excluded from the study.

*Trauma-Exposed (TE)* individuals were participants who were exposed to a traumatic event but did not meet diagnostic criteria for PTSD. They were included if they screened positive for Life Events Checklist but did not meet CAPS-5 criteria. This means that severity ratings for all categories were either absent (0) or mild/subthreshold (1) which are symptoms not severe enough to reach clinical significance. Any history of moderate-severe traumatic brain injury, psychosis, or other significant medical or mental disorders that may substantially influence cognitive performance excluded participation.

*Non-trauma-exposed (NTE)* individuals were included if they did not have a history of trauma exposure, moderate-severe traumatic brain injury, psychosis, or other significant medical or mental disorders that may substantially influence their cognitive performance. Exclusion criteria included adults unable to consent and any subject with BAI (≥19) or BDI-II (≥20) scores, indicating a moderate level of severity or greater. Participants must also be native or fluent English speakers, for the same reason mentioned above. Table 4 below elaborates on more information about participants in this study.

**Table 4:** Demographic information.

**Participants**	**Age**	**Sex**	**BAI**	**BDI-II**	**TOPF (SS)**
**Non-trauma exposed**					
1^*^	31	M	2	2	119
2^*^	25	M	5	2	106
3	22	M	8	9	109
4	25	F	0	1	98
5	20	M	0	0	99
6	29	F	6	10	115
7^*^	29	M	10	7	113
8^*^	20	F	10	17	98
9	34	F	1	0	101
10^*^	20	F	3	11	103
11^*^	23	F	6	6	85
Mean	25.2		4.6	5	104.2
MRI mean	24.6		6	7.5	104
**Trauma-exposed**					
1^*^	33	F	0	1	111
2^*^	23	F	7	22	114
3	52	M	25	20	96
4^*^(MRI excluded due to artifacts)	34	F	42	37	104
5	30	M	34	35	116
6	18	F	9	9	96
7	57	F	1	1	92
8	21	F	4	3	87
9^*^	37	M	1	1	116
10^*^	47	F	1	0	115
11	33	F	13	1	119
12^*^	25	M	17	11	113
13^*^	20	M	3	3	115
14^*^	61	M	0	0	73
15^*^	32	M	0	4	104
16	59	M	4	9	119
Mean	36		10	10.4	105.6
MRI mean	34.7		3.6	5.2	107.6

^*^Refers to individuals who had MRI's conducted.

## Neuropsychological procedures

### Novel measures

Two novel computerized tasks were administered, including a measure evaluating capacity for Mental Time Travel (MTT; i.e., autobiographical recall and prospection) and a self-referential task (SRP). The MTT measure consisted of 30 words controlled for frequency, valence, and difficulty level. Participants were first presented with instructions and, upon verbal acknowledgment of understanding, were provided with two sample trials. This was followed by formal testing with a 1-min response time limit. The participant viewed a word displayed on the computer screen, and when a memory or prospective experience was rendered, the subject clicked the spacebar. This led to another display simply stating “elaborate,” during which subjects verbalized the memory or prospective event, and reaction time was measured. This was audio recorded and scored for instruction coherence, degree of detail, temporality (i.e., past vs. future), and neutrality. Following the computer test, three Likert scale questions were presented for participants to answer regarding perceived vividness, valence, and temporality.

Investigations of SRP are commonly performed using statements that describe a subject's own personality, attitudes, or preferences as cues. Our novel measure of SRP consisted of three phases. In the first phase, subjects were presented with a series of 50 personality traits derived from the Big 5 (10 traits from each category), based on the five-factor personality model (Goldberg, 1992), with items extracted and adapted from the International Personality Item Pool (Goldberg et al., 2006). Half of the words were representative of each category, whereas the other half were in opposition. Each word was presented with a threshold (i.e., 500 ms) to capture pre-reflective processes. Subsequently, they were prompted to answer “Agree” or “Disagree” and given 5 s to respond. This was ample time, as most individuals will respond to personally relevant information in < 3 s. For instance, in a prior study examining personally relevant information, the control group responded within 2,310 ms (Bluhm et al., 2012). Here, we measured reaction time and congruence with personality disposition to evaluate the self-referential effect, whereby individuals display improved memory for personally relevant words compared to non-relevant words. This categorization of items opens another channel of investigation, whereby different personality constellations can be associated with resilience and other factors. Moreover, too many incongruent responses within the same category can be indicative of poor effort. In the second phase, subjects were presented with 50 general, non-self-related items (e.g., box, wire, key, etc.). To quantify the self-referential effect, we introduced a third phase, which included an incidental recall task. Subjects were presented with all words from the prior two phases, including 25 distractor words for each phase. The SRE was calculated by dividing the number of words recalled from phase 1 by words recalled from phase 2. If distractor words were recalled, these were subtracted from their total number respective to each phase.

### Existing measures

Relevant measures include: The ER-40, The Hinting Task, and the Mind-Wandering Questionnaire. The Penn Emotion Recognition Task (ER-40) is a measure of social cognition, examining facial recognition of emotions. In particular, participants are sequentially presented with 40 colored photographs of individuals expressing different emotions and emotional intensity (e.g., sadness, anger, neutral, fear). Age, ethnicity and gender are balanced for each emotion. Scores account for accuracy and reaction time.

The hinting task is also a measure of social cognition, specifically examining representational theory of mind. It consists of 10 short passages with characters who are in dialogue with one another. One of the characters is indirectly making suggestive comments, and the participant is then asked what it is they are indirectly attempting to communicate.

The mind-wandering questionnaire that examines the extent to which an individual experiences mind-wandering on a daily basis. It is commonly used for quantifying mind-wandering and well-validated (Mzarek et al., 2013).

## Imaging procedures

### Whole brain segmentation

Participants underwent neuroimaging using a 3T MRI scanner (Skyra, Siemens Healthcare). Brain parcellation was performed using a 3D T1-weighted MPRAGE sequence with 1.0 mm isotropic resolution, a repetition time of 2,530 ms, an echo time of 3.3 ms, an inversion time of 1,100 ms, and a flip angle of 7°. Cortical and subcortical regions of interest (ROI) were analyzed using the standard pipeline of FreeSurfer (Version 6.0). The processing steps included motion correction, removal of non-brain tissue with a hybrid watershed/surface deformation algorithm, automated Talairach transformation, segmentation of subcortical white matter and deep gray matter structures (e.g., hippocampus, amygdala, caudate, putamen, and ventricles), intensity normalization, tessellation of the gray/white matter boundary, automated topology correction, and surface deformation guided by intensity gradients to refine gray/white and gray/cerebrospinal fluid boundaries.

Volumes and thicknesses of cortical gray matter (GM), white matter (WM), and key cognitive regions were quantified using FreeSurfer 5.3 (http://surfer.nmr.mgh.harvard.edu) with subcortical segmentation and cortical parcellation conducted according to the Desikan-Killiany atlas (Desikan et al., 2006). The volumes and thicknesses of cerebral GM and WM regions and the cognitive-critical regions were measured automatically with FreeSurfer 5.3 (http://surfer.nmr.mgh.harvard.edu) to perform subcortical segmentation and cortical parcellation according to the Desikan-Killiany atlas (Desikan et al., 2006).

ROI's included: medial prefrontal cortex, posterior cingulate cortex, anterior cingulate cortex, precuneus, bilateral temporoparietal junction, hippocampus, parahippocampal regions, and thalamus.

### Hippocampal subfields

Brain parcellation and hippocampal subfields (HSs) were obtained using a three-dimensional (3D) T1-weighted magnetization-prepared rapid gradient-echo (MPRAGE) sequence with 1.0 mm isotropic resolution, as well as a dedicated 3D T2-weighted sequence with an in-plane resolution of 0.4 × 0.4 mm^2^ and 2-mm slice thickness, respectively. Other imaging parameters of the T2 sequence included repetition time/echo time (TR/TE) of 8,540/80 ms and flip angle of 150°. The MRI data were acquired using a 3T scanner (Skyra, Siemens Healthineers). The HSs segmentation utilized both the high-resolution T2- and the T1-weighted images using the Automatic Segmentation of Hippocampal Subfields (ASHS) tool (Yushkevich et al., 2014), which combines multi-atlas label fusion and learning-based error correction guided by the “UPenn PMC Atlas.” Each hippocampus was segmented into five HSs: CA1, CA2, CA3, DG and the subiculum on T2-weighted images. Total hippocampal volume, measured using the T2-weighted images, is the sum of the HS volumes.

## Results

### Neuropsychological differences between trauma-exposed and non-trauma-exposed groups

**Figure 2:**
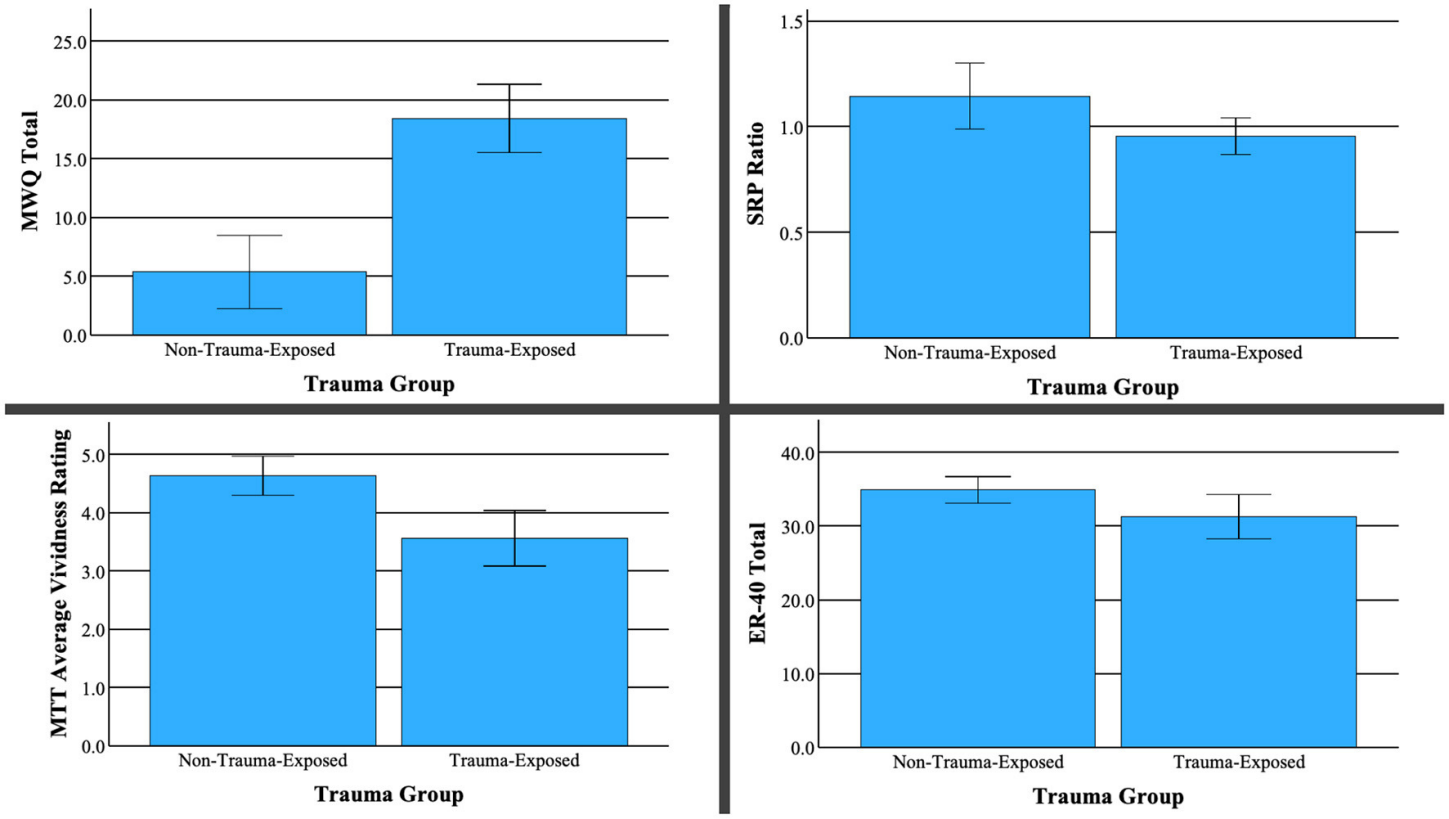
Differences between trauma-exposed and no trauma groups on neuropsychological measures.

Compared to the non-trauma group, TE participants displayed a lower ratio of true trait responses relative to true semantic responses (controlling for responses to distractor adjectives), [t_(25)_ = −2.48, p = 0.020]. This difference indicates lower self-referential processing for the TE group relative to the NTE group. Additionally, individuals from the TE group reported significantly lower vividness of visual imagery from the MTT measure, [t_(25)_ = −3.97, p = < 0.001]. Notably, ratings of event valence from this same task were statistically equivalent across groups, t_(24)_ = 1.864, p = 0.075. The TE group also scored significantly higher on the MWQ, [t_(31)_ = 6.683, p < 0.001], indicating an increased propensity for mind wandering. Finally, TE subjects performed worse at facial recognition than NTE subjects on the ER40 [t_(24)_ = −2.24, p = 0.018]. No other neuropsychological differences were found between the TE and NTE groups. Bar graphs depicting significance difference between TE and NTE groups are shown in Figure 2.

### Associations between neuropsychological tests

**Figure 3:**
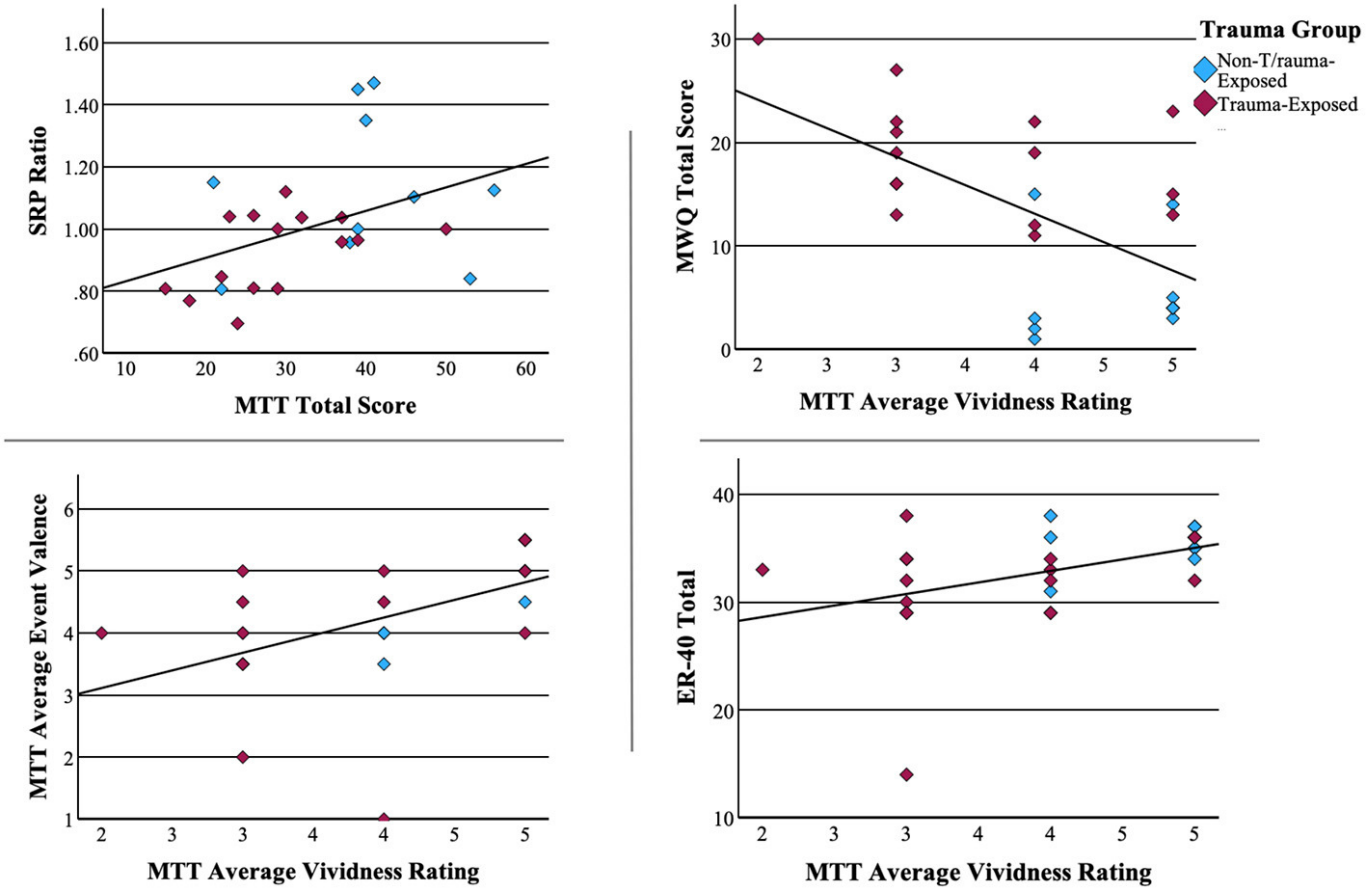
Associations between neuropsychological measures. Pearson correlations of significant associations between neuropsychological measures.

Vividness ratings from the MTT demonstrated a moderate negative correlation with mind wandering [r_(25)_ = −0.612, p < 0.001]. Mind wandering also correlated positively with the Beck Depression Inventory at the level of a trend [r_(25)_ = 0.355, p = 0.075]. Vividness was moderately correlated with average event valence [r_(25)_ = 0.503, p = 0.009], indicating an increased frequency of positive recollections, and with scores on the ER40 [r_(25)_, = 0.410, p = 0.038], suggesting an increased ability to recognize emotions. Finally, self-referential effect and MTT total score correlated positively [r_(25)_ = 0.417, p = 0.038]. This is consistent with the well-established efficiency of self-related information processing. Scatterplots of significant associations between neuropsychological measures can be found in Figure 3.

### Neural differences between TE and NTE groups

Unexpectedly, no volumetric differences among any of the regions of interest were found when comparing the TE and NT groups.

### Associations between neural volume and neuropsychological performance

**Figure 4:**
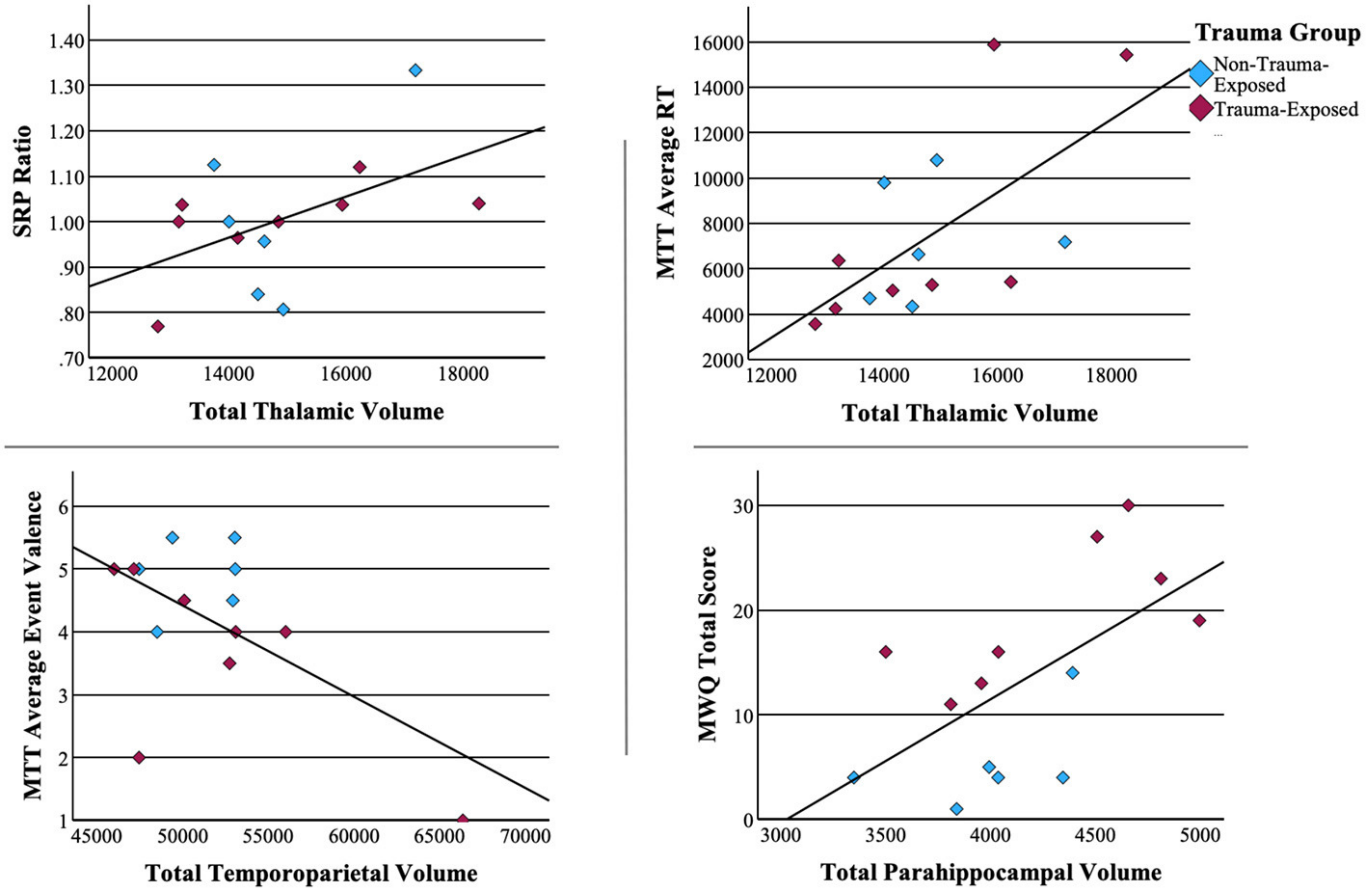
Associations between neuropsychological measures and brain volume. Pearson correlations of significant associations between neuropsychological measures.

**Figure 5:**
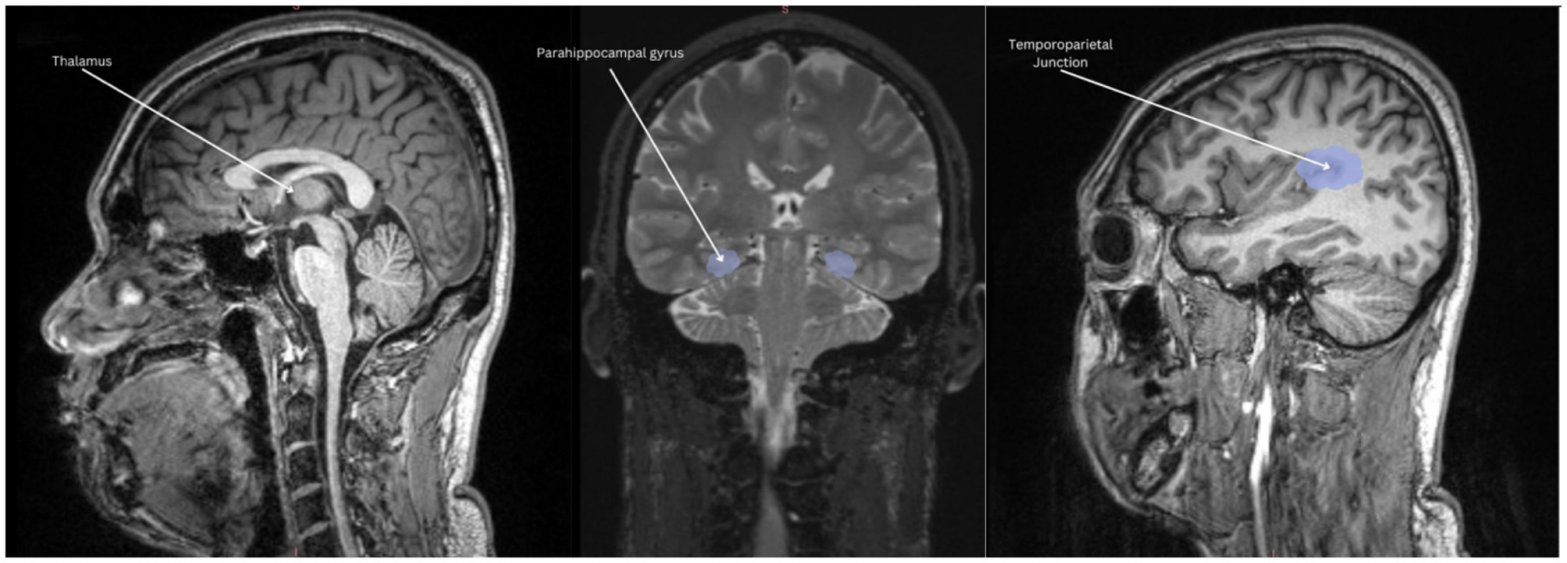
Volumetric associations to neuropsychological performance. Thalamic volume positively correlated with mental time travel reaction time and self-referential effect. Parahippocampal volume positively correlated with increased mind-wandering and depressive symptoms. Temporoparietal volume negatively correlated with the amount of positive events retrieved.

To better understand the potential neural differences underlying performance on neuropsychological measures, we examined correlations between our neural regions of interest and neuropsychological measures that differentiated the TE vs. NTE groups. These tests revealed a trend-level positive correlation between the self-referential effect and total thalamic volume [r_(12)_ = 0.501, p = 0.068]. Similarly, MTT average reaction time strongly correlated with thalamic volume [r_(12)_ = 0.633, p = 0.015]. Mind wandering had a strong positive correlation with parahippocampal volume [r_(12)_ = 0.618, p = 0.018]. Finally, average event valence (i.e., negative, neutral, positive) during memory retrieval or future event simulation had a moderately negative correlation with temporoparietal region volume r_(12)_ = −0.581, p = 0.029. No other correlations reached statistical significance. Scatterplots of significant associations between neuropsychological performance and brain volume can be found in Figure 4. Figure 5 depicts MRI images identifying the three main regions (highlighted in blue) where significant correlations were found.

## Discussion

In sum, our findings support previous research suggesting that trauma may dysregulate DMN functioning. These include reductions in (1) self-referential effect, (2) emotion recognition, (3) image vividness, and (4) increased mind-wandering. This study also encountered potential functions that were conserved in TE demographic, specifically in relationship to the specificity of detail included in the recounting of prospective events and memories, as well as the proportion of negative to positive events shared. Findings related to structural data suggests that the conservation of DMN integrity may be a protective factor for PTSD. Indeed, reduced hippocampal volume has been posited to be a risk factor (rather than consequence) for PTSD, with conserved or greater volumes related to resilience. This is in support of extant literature (Roeckner et al., 2021). Finally, reduced cognitive features from trauma exposure is more likely to be reflected in function rather than neural structure.

The trauma-exposed group demonstrated a reduced self-referential effect (SRE). Reduced ability to distinguish between self-related stimuli and impersonal data may correspond to an objectification of oneself. This aligns with those suffering from PTSD who may exhibit reduced SRE, suggesting a conflation between self-object (Lanius et al., 2011). Trauma may lead to an experience of dehumanization (Frewen and Lanius, 2015) and the reclaiming of selfhood is typically a goal in psychotherapeutic interventions related to trauma. From another perspective, reduced self-referencing as a defense against an on-going trauma may mitigate the magnitude of emotional experiencing, which may have been adaptive.

Social cognitive deficits, in particular reduced emotion recognition relates to a decreased ability to accurately read facial expressions. This may contribute to disruptions in interpersonal relationships commonly seen in traumatized individuals. Ambivalence, which may arise due to incongruent feedback from continuous misinterpretations of facial cues, may also sustain hypervigilance, biasing individuals toward decontextualized protective behaviors, such as avoidance.

While research demonstrates greater vividness in recollections of traumatic memories (Schönfeld and Ehlers, 2017), and reduced vividness and specificity in general memories and prospective imagery in individuals with PTSD vs. control (Brown et al., 2013); such levels in trauma-exposed groups without PTSD have yet to be examined. Our study indicates that while the vividness of mental imagery related to memory recall and prospection is reduced in trauma-exposed demographics, the specificity of recounted memories did not change. Moreover, the healthy sample and trauma-exposed sample were statistically equivalent when comparing the amount of positive vs. negative memories/projections. These may provide deeper insight into functions that might distinguish between trauma-exposed and those with PTSD, with these conserved elements being protective.

Longer reaction times in the retrieval of memories and/or projection of future scenarios may be a form of selectiveness, that may be adaptive in the individual's capacity to maintain stability. That this has been linked to a greater volume in the thalamus at a trend-level is not surprising considering previous research. Beyond the role as a sensory relay station, the thalamus has been implicated as a major passage of metabolic activity fundamental to its connectivity with other areas of the DMN (Alves et al., 2019). In this context, it is intuitive to find that greater thalamic volume correlated to improved self-referential processing. Moreover, the thalamus can be viewed as a toggle between mindfulness and mind wandering. Specifically, *reduced* thalamic activity is more correlated to mindfulness (Wang et al., 2014, p. 1), which may relate to a greater thalamic volume because it can afford improved efficiency.

The trauma exposed group presented with increased mind wandering, which itself, has been positively correlated to negative emotions (Killingsworth and Gilbert, 2010), and this was further supported in our study through positive correlations between mind wandering and the Beck Depression Inventory—Second edition. Moreover, consistent with extant literature, a positive correlation was identified between mind wandering and parahippocampal activity and reduced vividness related to higher levels of mind-wandering. What is unique in this study, is the finding that there was an equivalence of proportion of negative to positive mental events from recall or prospection found between NTE and TE groups. Considering that the TE group appeared to mind wander more, there may be further differences to be uncovered between externally elicited actions from the DMN (i.e., intentional autobiographical recall, prospection) and unintentional internal resting state manifestations.

This study confirms no significant changes in hippocampal subfields, such as the dentate gyrus, which has been found to be smaller in those at higher risk for developing PTSD (Koch et al., 2021). As mentioned earlier, it may be the case that the TE group was partially protected by their healthy hippocampal size. Finally, we found a negative correlation between the temporal parietal junction (TPJ) and event valence, suggesting that as the volume of the TPJ increased, so did the likelihood of negative valence appear. This might relate to higher levels of mind wandering and rumination about potential negative events.

## Future directions and limitations

The most salient limitation of this study is the low sample size. It will be of benefit for future studies to examine a larger number of control and trauma-exposed subjects to ensure that these findings may be replicated. With sufficient resources, including neuropsychological and brain imaging data from a PTSD group would greatly enhance the viability of this studies objective, providing further specification into what variables may have been protective against PTSD pathology. As an example, evaluating the effects of PTSD while controlling for the effects of trauma exposure would yield a much more nuanced data set. This study was also too small to distinguish amongst varying responses within the category of trauma-exposed without PTSD. As an example, there are those who may have maintained a healthy level of functioning without symptoms and/or those who experience post-traumatic growth. It would be wise for future studies to include measures of resilience to provide even greater elaboration and understanding of how these groups may differ rather than combining them.

Furthermore, a deeper level of analysis examining how regions and functions of the DMN relate to factors of resilience would have provided a greater understanding that may be of clinical use. Regarding the tools being used, creating a separate measure for prospection and autobiographical recall may provide an enhanced understanding of how these overlapping but distinct functions may be impacted. Finally, gathering more data related to psychosocial history may provide a greater understanding of what some vulnerable and protective factors might be. Given the insignificance of volumetric differences in this study, future studies may yield more beneficial results by focusing on the functional differences.

Generally, there is a great lack of research concerning neuroimaging and trauma-exposed groups. While there are several limitations to this pilot study, our review indicates that it provides much-needed introductory work into research that may help the field of neuropsychology advance preventative measures, treatments, and assessments for trauma and PTSD.

## Data Availability

The raw data supporting the conclusions of this article will be made available by the authors, without undue reservation.

## Footnotes

^1^DMN integrity has been compromised in disorders such as mild cognitive impairment (Jacobs et al., 2013), schizophrenia (Whitfield-Gabrieli et al., 2009), traumatic brain injury (Bonnelle et al., 2011). Sandrone (2012) compared the DMN to a “crystal ball” identifying a whole host of other disorders whereby the DMN is thought to have predictive or diagnostic capacity, these include: juvenile offenders and impulsivity, early detection/ discrimination of Lewy body dementia from Alzheimer's disease, severity of multiple sclerosis, and diagnostic and prognostic metric for patients with autism.
